# Antipsychotics use in dementia: How safe are they?

**DOI:** 10.1192/j.eurpsy.2021.1134

**Published:** 2021-08-13

**Authors:** C. Lopes

**Affiliations:** Clínica 3, Centro Hospitalar Psiquiátrico de Lisboa, Lisbon, Portugal

**Keywords:** dementia, safety, Antipsychotics

## Abstract

**Introduction:**

Antipsychotics are frequently used for managing psychiatric and behavioral symptoms of dementia. However, it’s an off-label resource which remains controversial due to significant safety concerns in the elderly population, namely increasing cardiovascular adverse effects.

**Objectives:**

To access antipsychotic safety and potential risks when used in dementia.

**Methods:**

A non-systematic review was carefully conducted on PubMed using the following keywords: “dementia”, “antipsychotics” and “safety.” We selected clinical trials, meta-analysis, randomized controlled trials published in the last 10 years.

**Results:**

A total of 43 articles was obtained, of which 22 were excluded because they didn’t meet our inclusion criteria. Regarding atypical antipsychotics, one study found an incidence of severe events in 23,7% of patients and a OR=2.5 for cerebrovascular side effects. Quetiapine was suspended midway given it had a higher incidence of adverse effects compared to others. There weren’t any significant statistic differences concerning serious events between classes of antipsychotics(p<0,01). No study was found comparing typical and atypical antipsychotics safety in dementia.

**Conclusions:**

Overall, we can conclude that antipsychotics pose a risk of serious adverse effects when used in elderly patients, namely cerebrovascular events. Among atypical antipsychotics, quetiapine, used frequently for controlling neuropsychiatric symptoms in these patients appears a higher risk for severe adverse events compared with other drugs. Their use should be restricted after there aren’t any other options available. New protocols could be developed to control these symptoms, for example, environmental measures before resorting to antipsychotics.

